# Methodology to evaluate stress corrosion cracking in ethanol environments, applied to circumferential welds on API 5 L steel pipelines

**DOI:** 10.1016/j.mex.2022.101675

**Published:** 2022-03-25

**Authors:** Elielson A. Santos, Vinícius Giorgetti, José B. Marcomini, Marcos R. Monteiro, Andrea M. Kliauga, Vitor L. Sordi, Carlos A.D. Rovere

**Affiliations:** a Graduate Program in Materials Science and Engineering, Federal University of São Carlos, Rodovia Washington Luiz, Km 235 SP 310, São Carlos, São Paulo 13565-905, Brazil; b Munir Rachid Corrosion Laboratory, Department of Materials Engineering, Federal University of São Carlos, Rodovia Washington Luiz, Km 235 SP 310, São Paulo 13565-905, Brazil; c Saudi Aramco, Dhahran, Saudi Arabia; d Department of Materials Engineering, University of São Paulo, Avenida João Dagnone, 1100, São Carlos, São Paulo 13563-120, Brazil; e Department of Mechanical Engineering, Federal University of São Carlos, Rodovia Washington Luiz, Km 235 SP 310, São Carlos, São Paulo 13565-905, Brazil

**Keywords:** API X70 steel, Fatigue crack growth, Stress corrosion cracking, Welded joint, Ethanol environment

## Abstract

This work presents an experimental methodology developed to perform fatigue crack growth (FCG) and slow strain rate (SSR) tests in ethanol environments aiming to evaluate stress corrosion cracking (SCC) susceptibility of circumferential welds on steel pipelines. FCG and SSR specimens were machined from a welded pipe and the notches were properly designed to promote crack propagation in the different regions of the weld. Tests were carried out keeping the crack-tip region fully immersed in an ethanol solution, which was fueled by a circulation system to ensure replenishment and aeration throughout the test. When applied to a welded API X70 steel pipe, this experimental methodology proved to be an efficient and simple method to achieve relevant and important informations on environmentally assisted crack growth and SCC susceptibility. The method developed here is inserted in the aspects as follows:

• Perform tests in slow strain rate and cyclic bend loading in circulating ethanol, to promote the fracture in the different regions of a circumferential weld joint of a steel pipe.
• Investigate sensitivity to stress corrosion cracking (SCC) of these different weld regions in ethanolic environment.
• This method presents constructive details of a suitable apparatus which the experiments can be easily replicated.

## Graphical abstract

**Figure:**
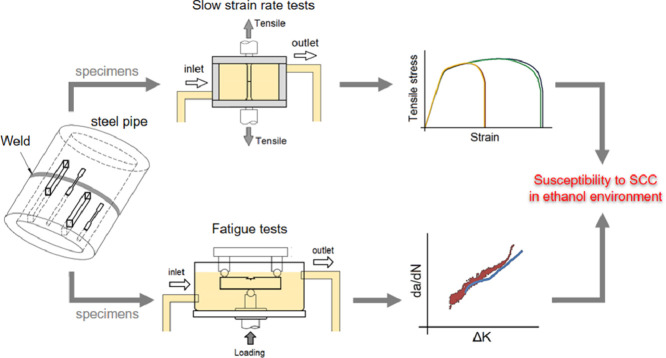

Specifications table

**Table:** 

Subject Area:	Engineering
More specific subject area:	Environmental Assisted Cracking (Stress Corrosion Cracking)
Method name:	Tests for environmental assisted cracking in ethanol environment
Name and reference of original method:	This method article makes mention to SSR and FCG tests performed in standard ethanolic solution reported on Ref. [5]
Resource availability:	No applicable

## Introduction

This article describes an experimental methodology to evaluate stress corrosion cracking (SCC) susceptibility of circumferentially welded steel pipes based on fatigue crack growth (FCG) and slow strain rate (SSR) tests performed in an ethanol environment. The effects of ethanol on the SCC behavior of API 5 L steels have been reported in the literature [1], [2], [3], [4], but the specific case of circumferential welds, such as those used in pipeline construction, has been largely underexplored [5].

SCC failures have been reported in carbon steels exposed to fuel-grade ethanol in field operations, including ethanol-carrying pipelines [4]. SSR test methods have been established for evaluating the SCC susceptibility of carbon steels in Fuel Grade Ethanol (FGE) and FGE-fuel blends, as well as synthetic ethanolic solutions prepared in laboratory [6]. According to ASTM G129 [7], SSR are not intended to necessarily represent service performance, but rather to provide a basis for screening, for environmental interaction with a material and for evaluation of effects of metallurgical and environmental variables on sensitivity to cracking. Otherwise, in the case of pipelines carrying hazardous liquid, cyclical loading arises from fluctuations in the internal pressure of the pipe, thus may result in crack growth due to fatigue condition [8], where FCG tests are more useful.

The experimental methodology described herein details a suitable apparatus which allows carrying out SSR and FCG tests properly designed to promote the fracture in the different regions of a circumferentially welded steel pipe.

### Welding procedure and design of specimens

**Fig. 1:**
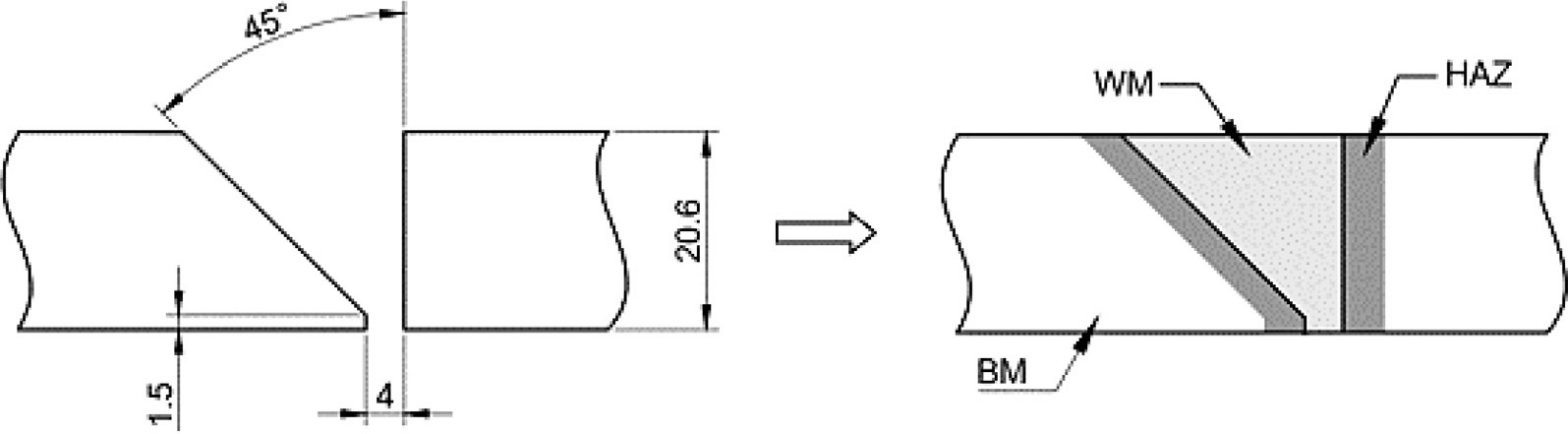
Dimensions (mm) of the single bevel groove used for the circumferential weld of an API 5 L X-70 steel pipe and respective regions of the welded joint.

**Fig. 2:**
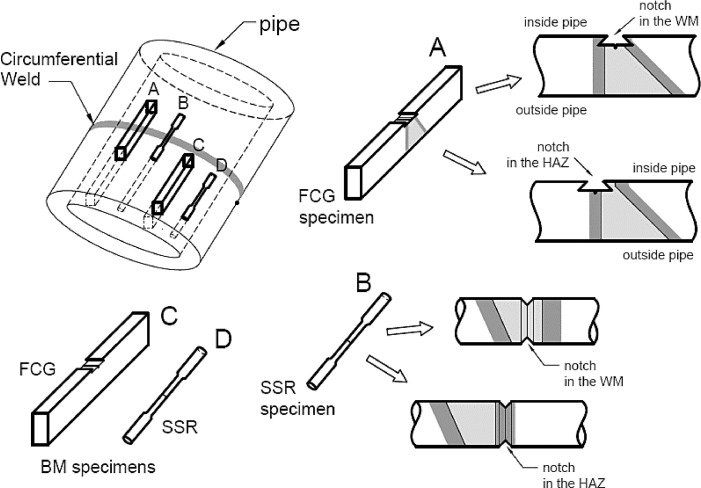
Schematic illustration of FCG and SSR specimens machined from the welded joint.

When developing the present methodology, samples of an API 5 L X70 [9] steel pipe, having 609.6 mm diameter (external) and 20.6 mm wall thickness, were joined by circumferential multi-pass welding following a standard procedure [10]. ER70S-3 electrodes were used for the TIG root procedure and E8010-G coated electrode as weld filler and finisher. A single bevel-groove (half V) with the dimensions shown in Fig. 1 was chosen to provide a more uniform heat-affected zone [5]. The specimens for FCG and SSR tests were properly designed and machined to promote crack growth in the regions of heat-affected zone (HAZ), weld metal (WM) and base metal (BM), as schematically illustrated in Fig. 2. The specimen's dimensions and testing procedures are detailed as follows.

### Preparation of a synthetic ethanolic solution

A standard synthetic ethanolic solution was prepared using the composition described below. According to NACE TM0111 [6], this solution, when aerated, has a high propensity to produce SCC in carbon steels.

• 3.75 vol% gasoline (ethanol-free).
• 1 vol% deionized water.
• 0.5 vol% methanol (CH3OH).
• 56 mg/L acetic acid (CH3COOH).
• 32 mg/L sodium chloride (NaCl).
• Balance - anhydrous ethanol 99.8% P.A.

It should be noted that this solution does not necessarily comply with the current compositional requirements defined by ASTM D4806 [11] for fuel grade ethanol. Since the susceptibility to SCC may be affected by ethanol chemistry, different formulations of fuel grade ethanol can be used to evaluate the effects of each chemical component [4,12].

Owing to the fact that, in Brazil, commercial gasoline contains ethanol additions, the following procedure was adopted to obtain an ethanol-free gasoline: distilled water was added to common gasoline (1 to 1 ratio) and was shaken vigorously, forming a heterogeneous solution consisting of two phases: gasoline (organic phase) and ethanol + water (aqueous phase). Subsequently, the aqueous phase was eliminated through a drain valve, thus obtaining ethanol-free gasoline. Sodium chloride (NaCl) was dissolved in deionized water and then acetic acid and methanol were added until complete dissolution of these elements. Later, this mixture was added to anhydrous ethanol and the recipient was shaken to mix of all components. Finally, the ethanol-free gasoline was added and mechanically agitated until complete homogenization, thus obtaining the synthetic ethanolic solution.

### Fatigue crack growth (FCG) tests

FCG tests were performed according to ASTM E647 [13], in air and in ethanol environments, using Instron® servohydraulic equipment coupled with da/dN software to obtain the crack growth rates as a function of the stress intensity factor variation (da/dN x ΔK curves).

**Fig. 3:**
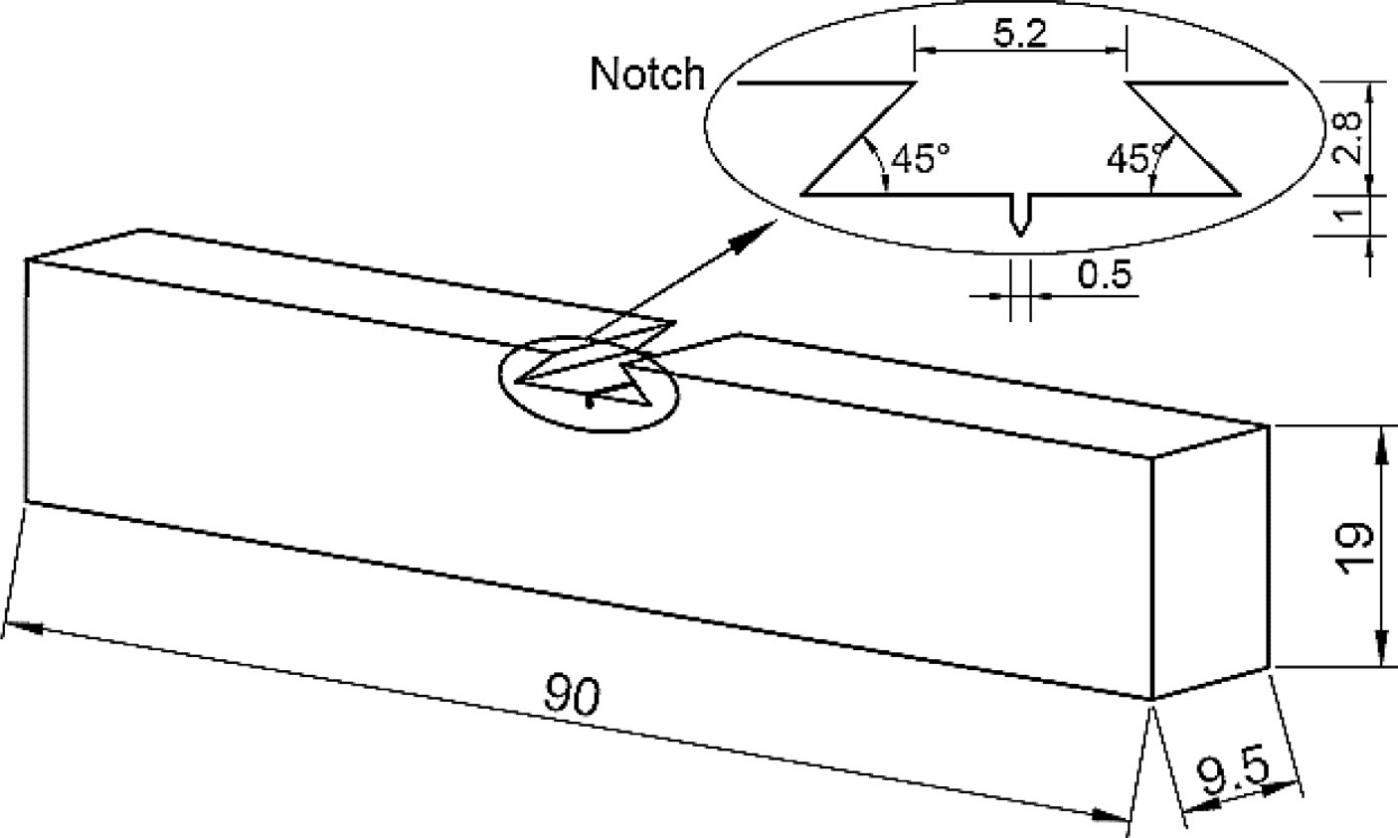
Standard three-point single edge notch bend, SEN B3, specimen. (Dimensions in mm).

**Fig. 4:**
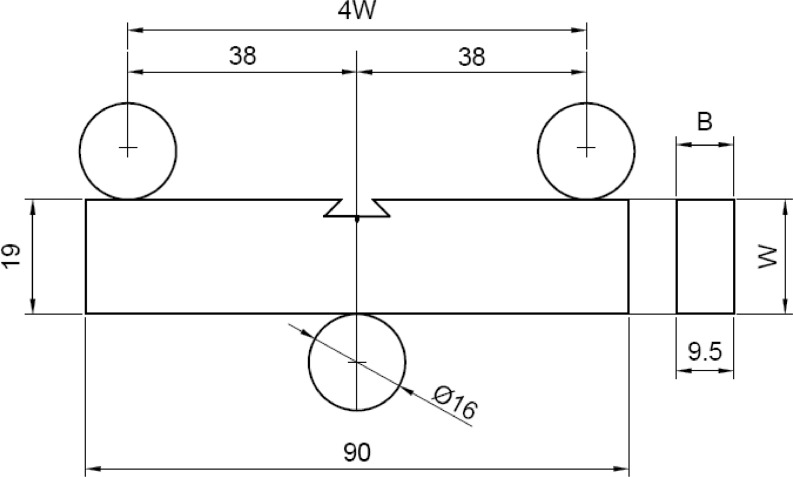
Fixture for tension-tension loading of a SEN B3 specimen. (Dimensions in mm).

The single edge notch bend, SEN B3, was the type of specimen used to carry out three-point bending FCG tests, in accordance with ISO 12,108 standard [14]. Specimens, whose dimensions are shown in Fig. 3, were machined from the welded pipe, and the notch was properly positioned in the different regions of the weld, namely WM, HAZ and BM, as shown in Fig. 2. Fig. 4 shows the loading fixture and respective dimensions. FCG tests were conducted at room temperature (∼25 ^o^C) under constant load amplitude using the compliance technique for crack size monitoring. A sinusoidal waveform was applied with a load ratio (R) equal to 0.1 and frequencies (f) of 15 Hz and 1 Hz for air and ethanol environments, respectively. The lower loading frequency was applied to enable corrosion to occur in the crack process, as it has been demonstrated that the crack growth rate under a corrosive environment may be affected by time-dependent phenomena [5,^13^,15].

In all cases, a fatigue pre-crack of about 1 mm in length was introduced at the notch tip in air environment, as recommended to eliminate mechanical notch effects [13,14]. The pre-cracks developed were checked to start the tests with the desired values of ΔK and initial crack size.

**Fig. 5:**
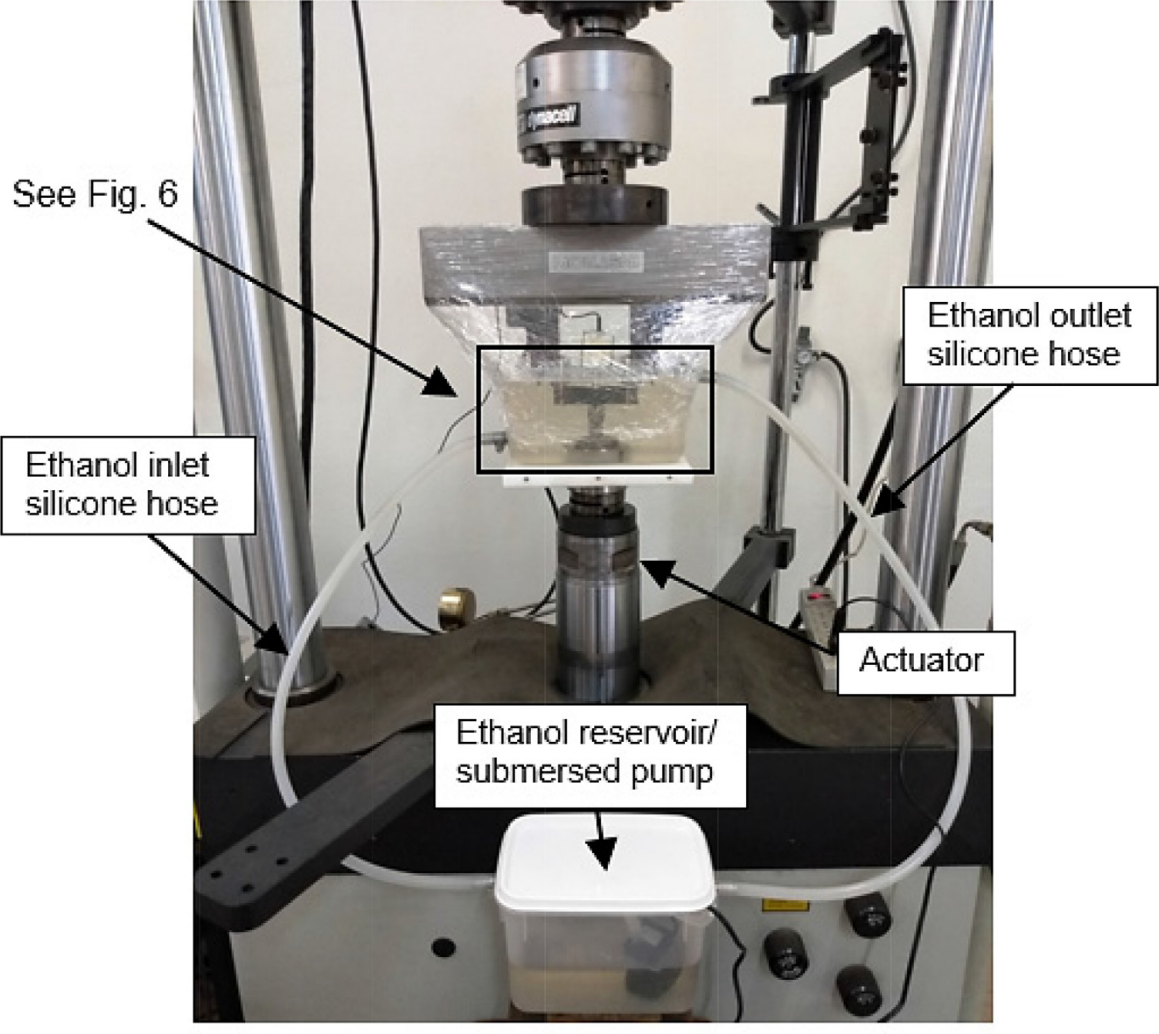
Experimental set up for FCG tests in an ethanol environment.

A circulation system was properly designed to provide a continuous flow of the test solution around the crack propagation region, which allows continuous aeration of the solution, promoting the removal of corrosion products [13]. Fig. 5 shows an overview of the testing apparatus and the circulation system, which includes: 02 (two) polypropylene reservoirs; 02 (two) silicone hoses; 01 (one) submersed pump (aquarium pump) and fixing accessories.

**Fig. 6:**
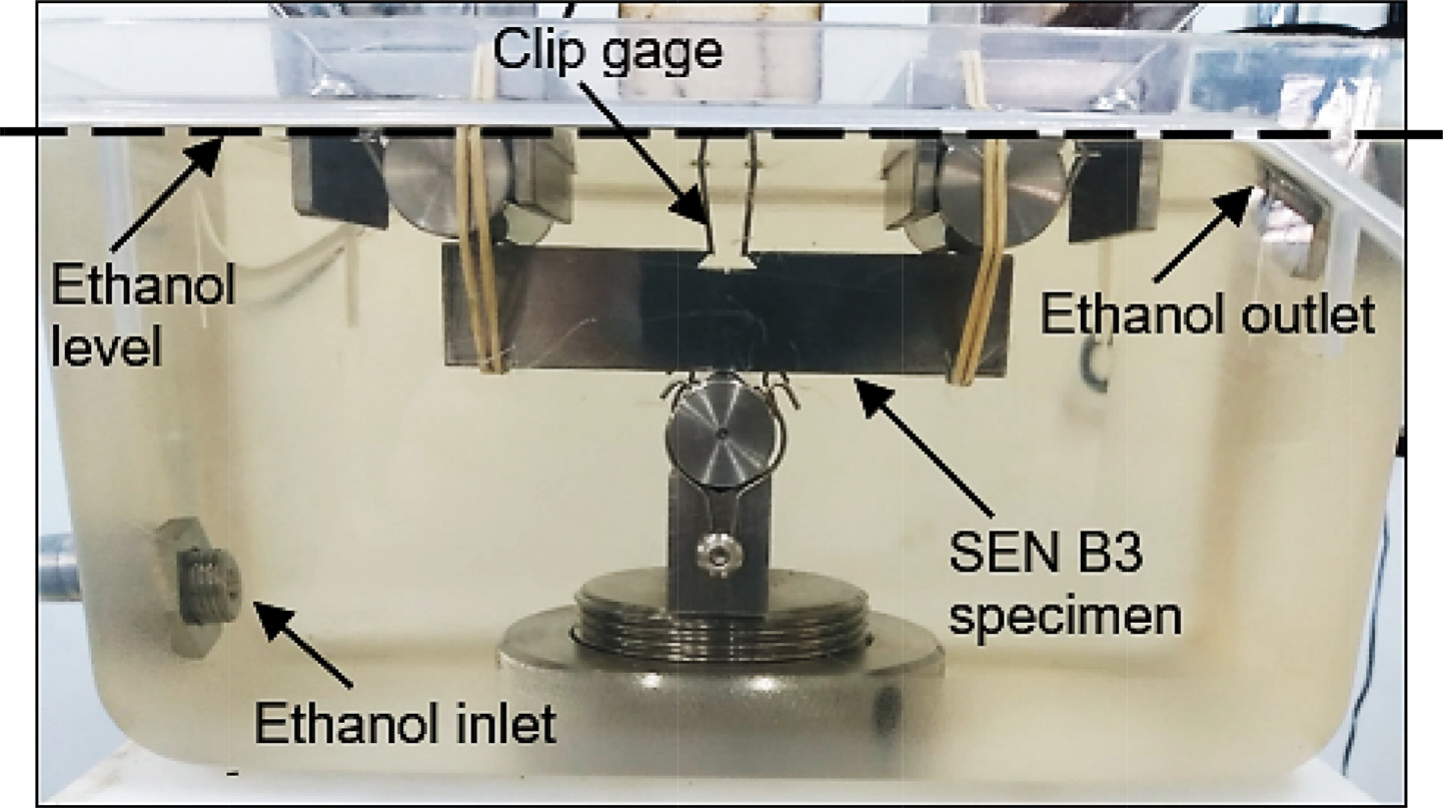
Reservoir with three-point bending assembly for FCG test with SEN B3 specimen immersed in ethanolic environment.

As can be seen in Fig. 5, the circulation system ensures the continuous replenishment of the reservoir that contains the specimen, maintaining the solution at a level such that the crack-tip region is fully immersed, while only rods of clip gage are reached by the liquid (see Fig. 6). Metal parts in contact with ethanol were made of stainless steel to avoid corrosion and contamination of the solution. A polyvinyl chloride (PVC) plastic film can be used to isolate the solution from the laboratory environment to minimize evaporation and/or humidity absorption. The specimen remains immersed throughout the test, which is performed at a loading frequency low enough to allow time-dependent corrosion processes to proceed.

### Slow strain rate (SSR) tensile tests

The SCC phenomenon was observed in several components of carbon steels in contact with ethanolic environments and extensive research has been conducted on the identification, repair and cracking mitigation of steel equipment in fuel ethanol services [4,16]. In this context, SSR tests with notched specimens, besides FCG tests, may provide rapid means to evaluate ethanol SCC in carbon steels [6,7].

In the present case, cylindrical notched specimens were properly machined from the welded pipe (see Fig. 2) to contain either WM, HAZ or BM at the center of the gage length, where the notch is positioned. SSR tests were performed at room temperature (∼23 ^o^C), in air and in ethanol, to compare the SCC susceptibility in the different regions of the weld. The nominal strain rate was set as 1 × 10–6 s^-1^, which means a constant ram speed equal to 1.52 × 10–3 mm/min, for the present specimen's dimensions.

**Fig. 7:**
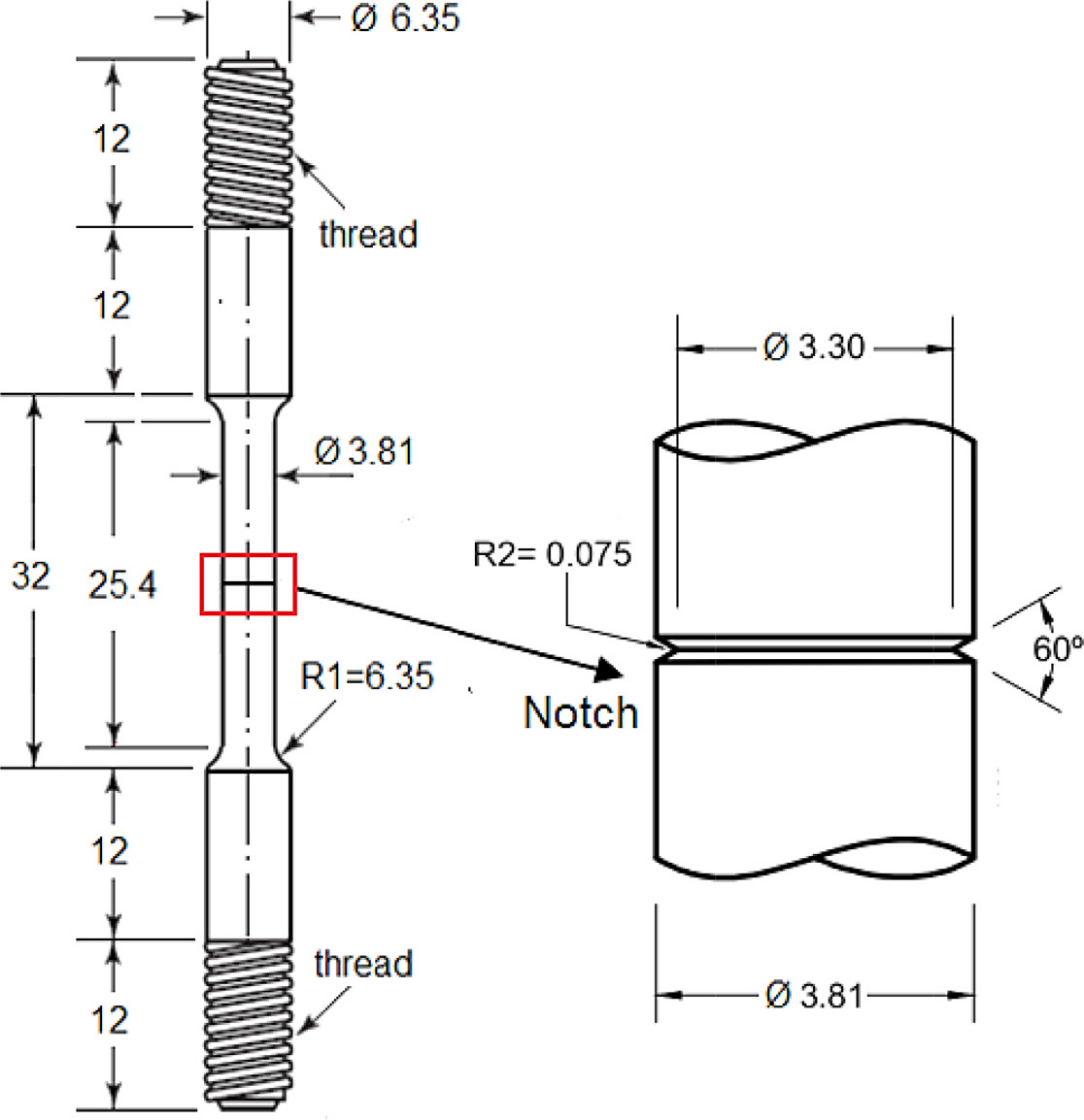
Specimen design for SSR tensile tests and notch geometry in detail. Dimensions in mm. Adapted from [6].

Fig. 7 shows the specimen´s design and notch details in compliance with the NACE standard TM0111 [6] requirements. The notch was machined using a special tool to meet the design desired measurements. Threaded ends were designed to clamp to the test machine claws, while smooth regions having the same a diameter fit the sealing rings of the environmental chamber.

**Fig. 8:**
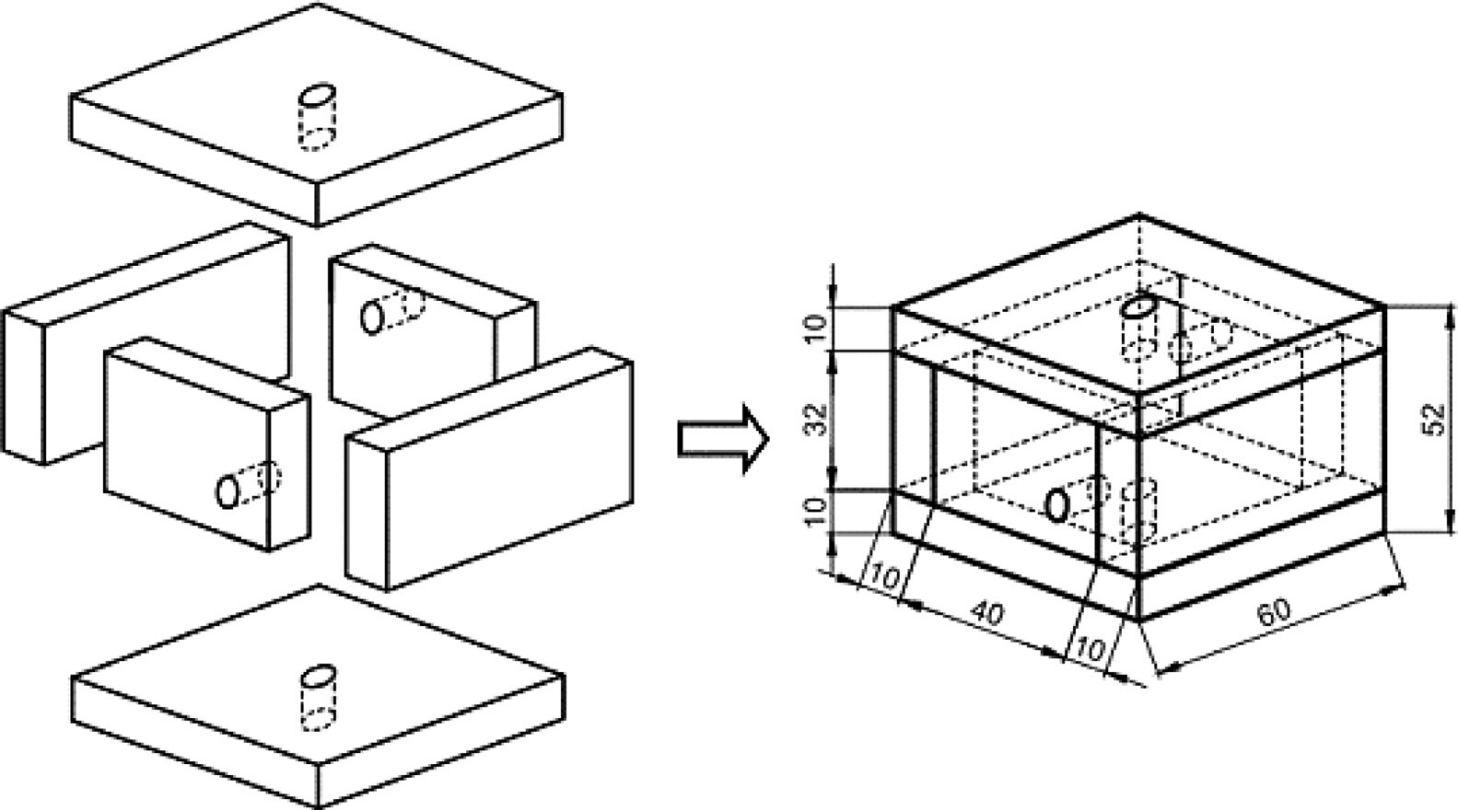
Schematic view of a polycarbonate chamber designed for SSR tests in an ethanolic solution. (Dimensions in mm).

**Fig. 9:**
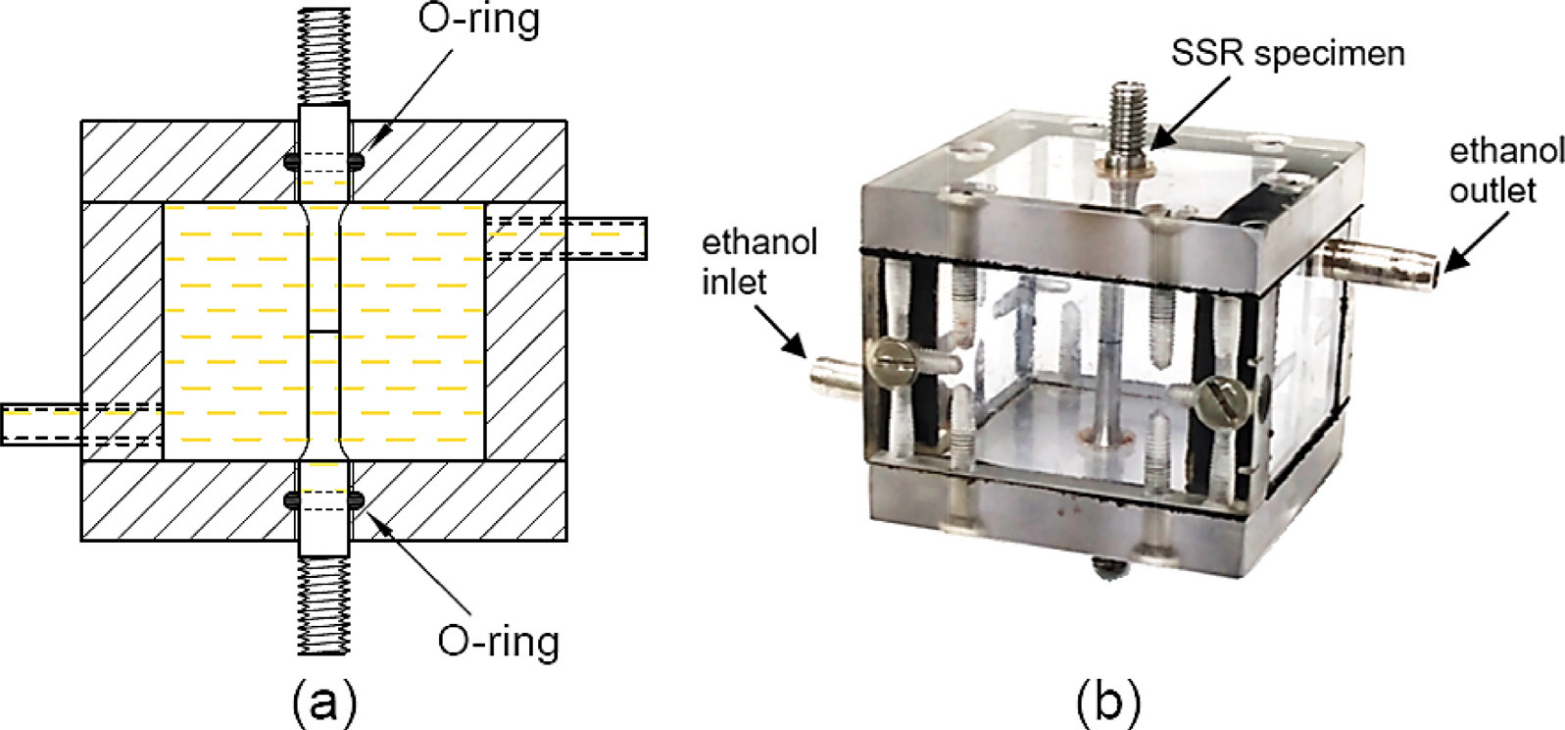
(a) Sectional view revealing the mounting of the seal O-rings. (b) SSR specimen assembled in the polycarbonate chamber with gage length inside and threaded tips outside the chamber.

A polycarbonate chamber was developed to allow the notched region of the specimen to be exposed to ethanol environment during the tests, avoiding the contact of the solution with the threaded-ends and grips. A schematic view of the chamber and constructive details are shown in Fig. 8. The chamber was constructed using 10 mm thick polycarbonate plates, which were fixed to each other by stainless steel screws [see Fig. 9(b)]. In order to prevent the solution from leaking during tests, synthetic rubbers of 1 mm in thickness were placed between the parts, while seal O-rings were fitted with the specimen in the upper and lower holes of the chamber, as can be seen in the sectional view of Fig. 9(a).

**Fig. 10:**
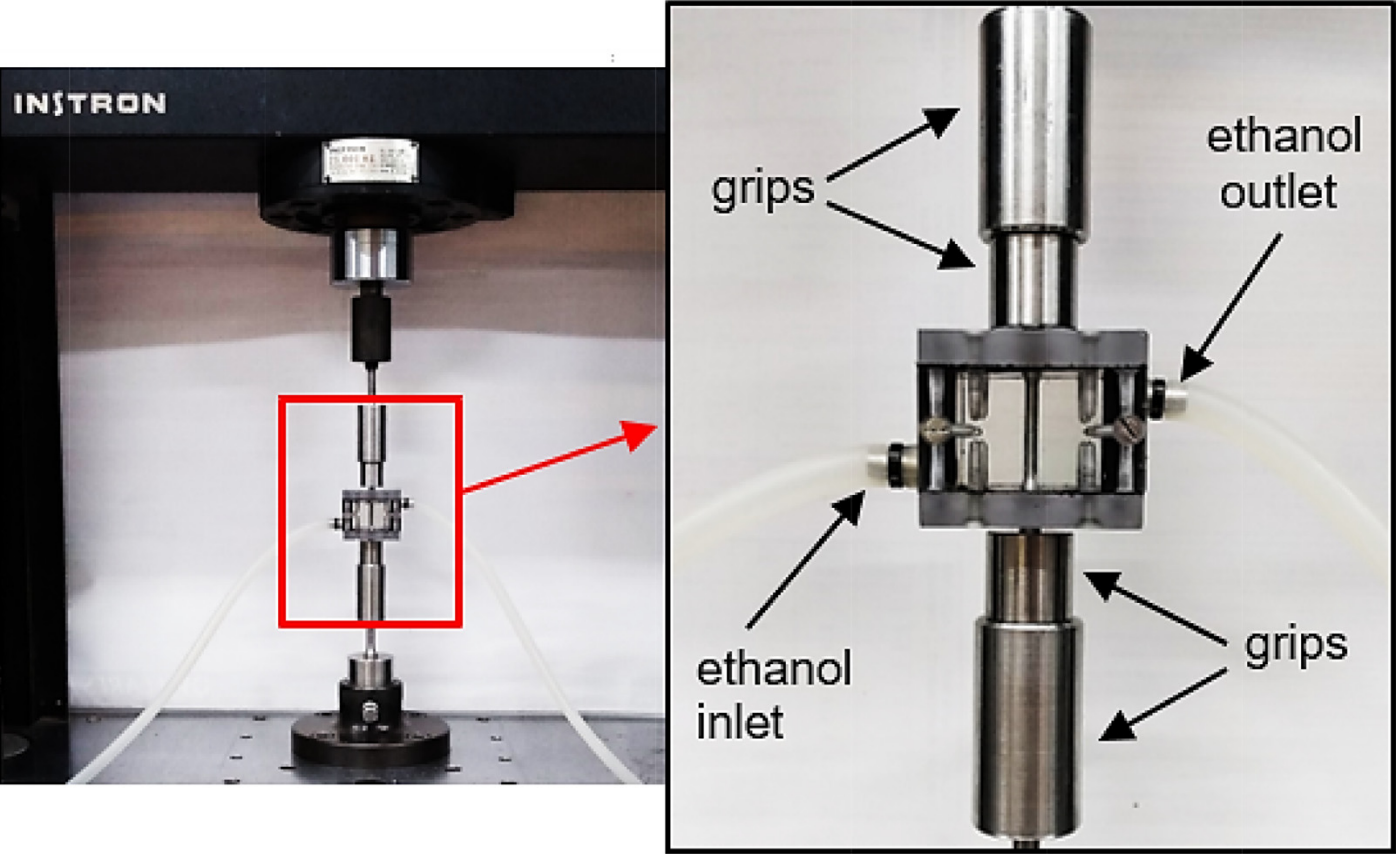
Experimental set up for SSR tests in ethanol environment.

Fig. 10 illustrates a universal testing machine assembled with the SSR device and a circulation system for the ethanolic solution, which is similar to that described for FCG tests. An enlarged detail shows of inlet/outlet hoses properly positioned so that the gage length of the specimen is fully immersed, keeping the notch in contact with the ethanol solution, while the threaded ends are placed outside the chamber.

## Method validation

**Fig. 11:**
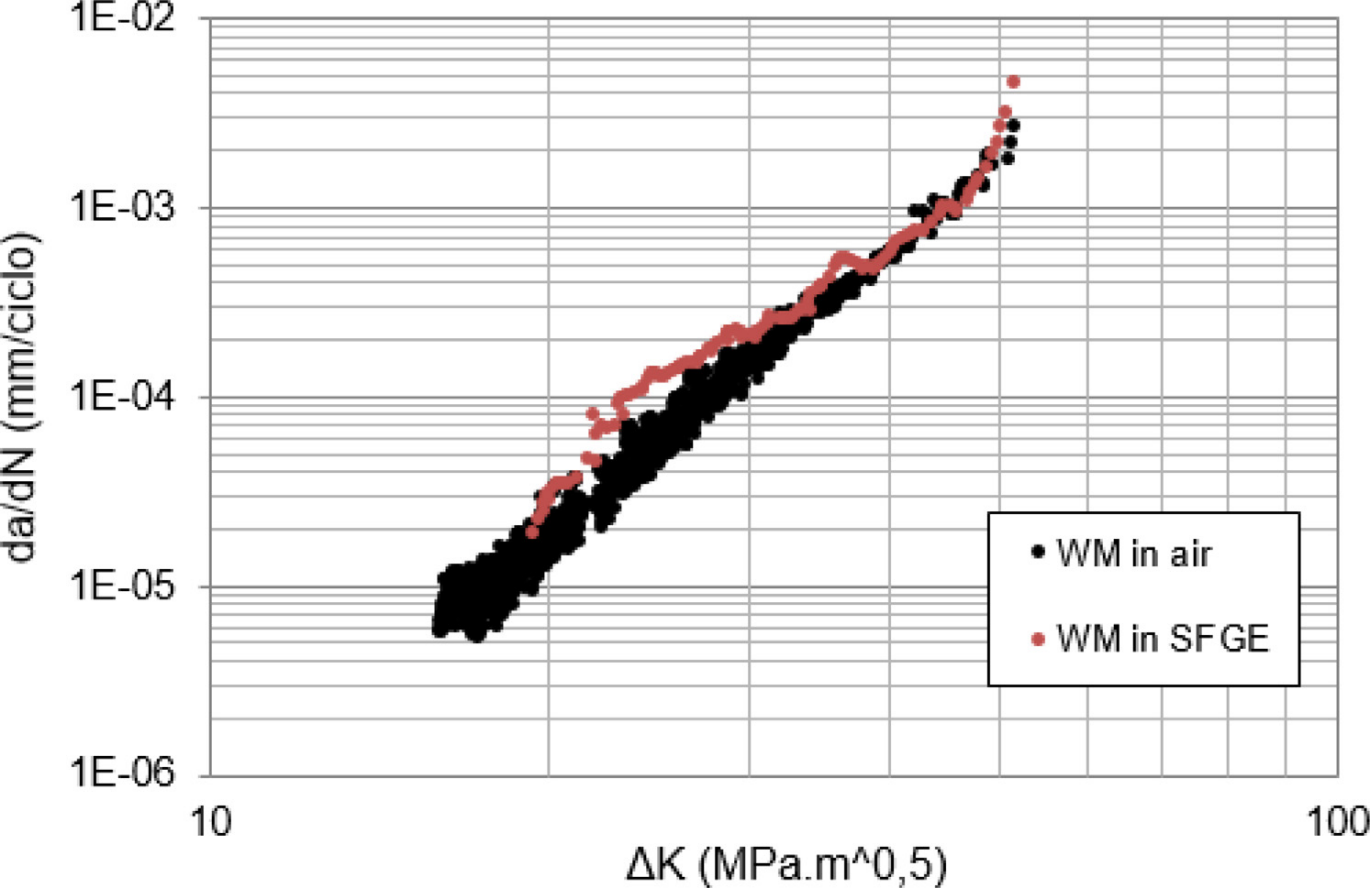
Fatigue crack growth rates as a function of ΔK through WM from tests performed in air and synthetic ethanolic solution. *R* = 0.1. *f* = 15 Hz (air) and *f* = 1 Hz (SFGE) [5].

**Fig. 12:**
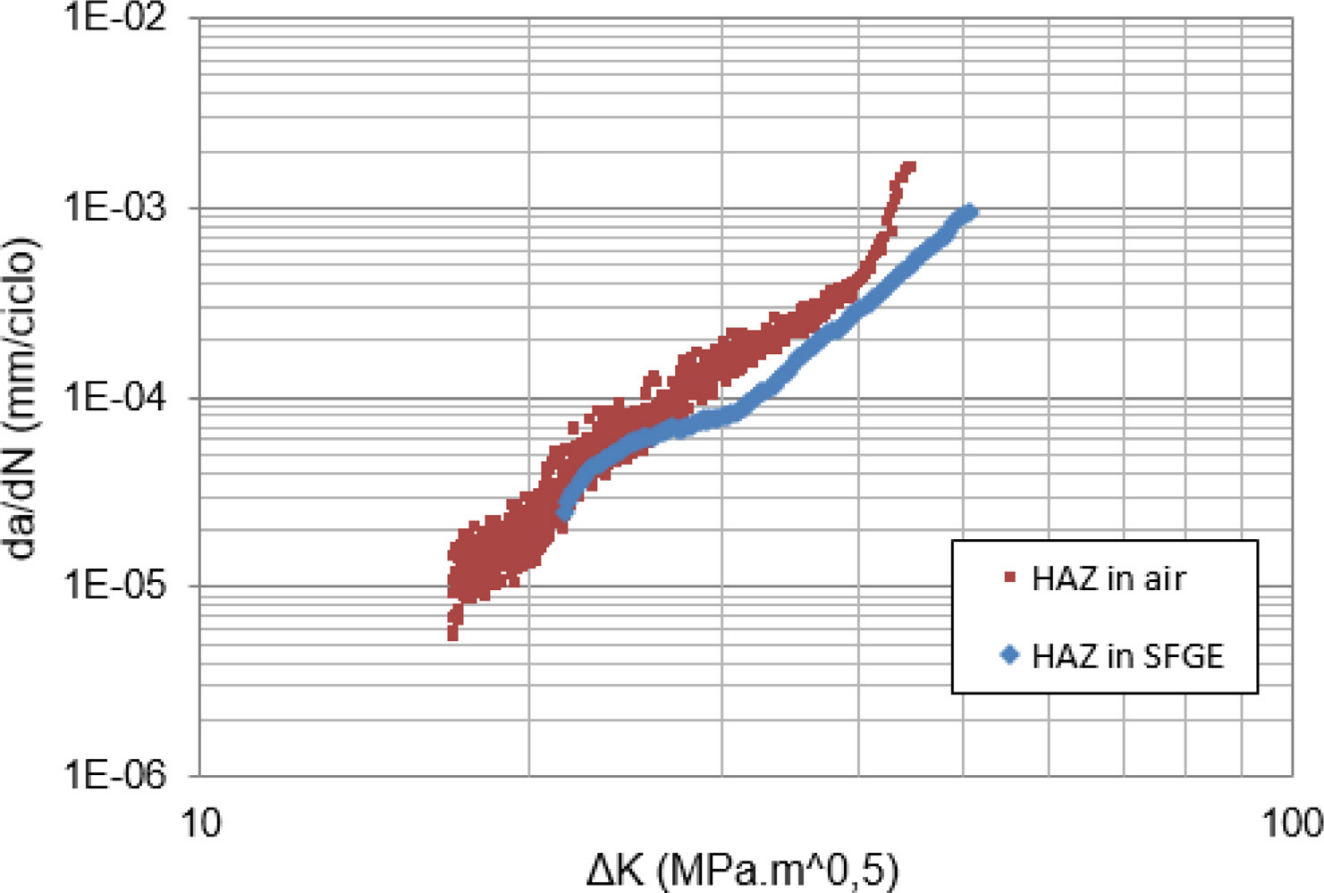
Fatigue crack growth rates as a function of ΔK through HAZ from tests performed in air and synthetic ethanolic solution. *R* = 0.1. *f* = 15 Hz (air) and *f* = 1 Hz (SFGE) [5].

The methodology described here was applied in a previous study [5] to evaluate the susceptibility of welded joints to SCC in a in a simulated fuel-grade ethanol (SFGE) environment, based on FCG and SSR tests. In that case, an SFGE was blended to produce the maximum impurity limits given in ASTM D4806 [11]. Fig. 11, Fig. 12 summarize the results from Ref. [5] in FCG tests designed to promote crack propagation, respectively, through the WM and through the HAZ of a circumferential weld on an API X70 steel pipe. The behavior of the curves (da/dN vs ΔK) was discussed to conclude that time-dependent corrosion fatigue interactions may involve alternating periods of increase and decrease in crack growth rates throughout the FCG tests. In this case, acceleration of crack propagation due to SCC predominated in WM and deceleration in HAZ [5]. Other authors [15,17] also observed the dependency of da/dN with the test frequency in ethanol environments, which was associated to crack-tip chemical reactions, including film rupture, passivation rate, solution renewal rate, as well as mechanical conditions and microstructure.

Regarding the SSR tests, in the study mentioned above [5] the present procedure proved to be adequate to assess the ethanol SCC susceptibility in different regions, i.e., HAZ and WM of a circumferential weld on an API X70 steel pipe. The occurrence of SCC was disclosed by a visible loss in ductility of the specimens tested in contact with ethanol (SFGE), which was an effect that was more evident for specimens having the notch positioned in the HAZ. In accordance, the fracture surface of these samples typically showed an external rim of SCC brittle fracture region. SSR tests using circumferentially grooved cylindrical specimens have been widely used to evaluate ethanol SCC in carbon steels [6,^7^,^18^,^19^,20], however, a specific methodology for circumferential field welds in steel pipes has not yet been systematically explored.

**Fig. 13:**
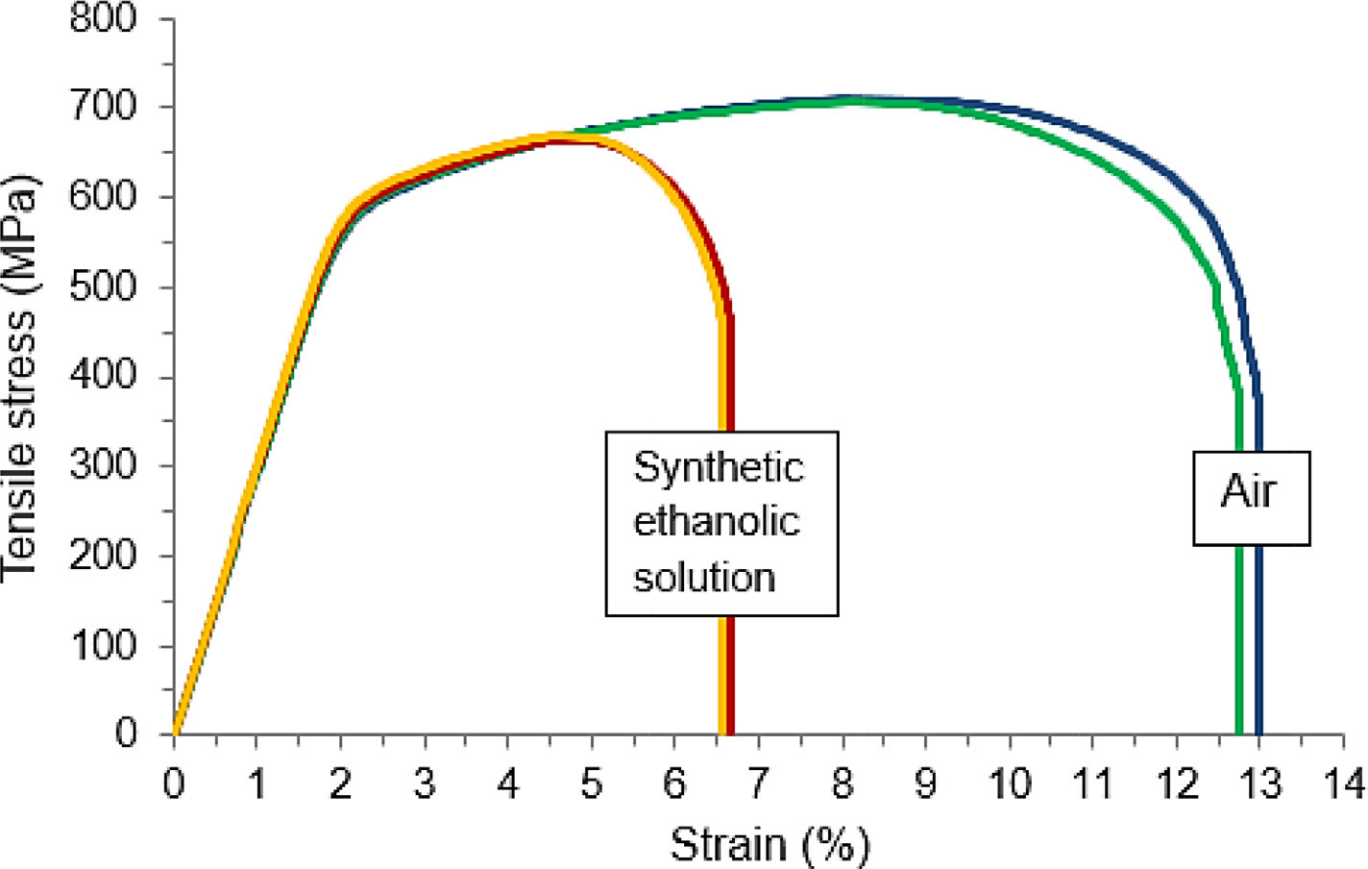
SSR tensile tests on cylindrical notched specimens of API X70 steel base metal (BM) in air and synthetic ethanolic solution environments.

Additionally, Fig. 13 presents the SSR tests performed on cylindrical notched specimens of an API X70 steel base metal (BM), using the experimental devices and procedures described herein. In this case a synthetic ethanolic solution was prepared using a composition suggested by NACE TM0111 [6], as already mentioned. As can be seen in Fig. 13, the curves obtained in the ethanol environment revealed a substantial reduction in the total strain to fracture, representing a loss in ductility of about 50%, if compared to the tests performed in air. The yield stress does not seem to have been affected by the presence of ethanol; meanwhile, an environment-induced cracking process clearly anticipated the fracture of the specimens, which reduced the strain hardening capacity and (consequently) the ultimate tensile strength. Other authors [3,18] also observed an important decrease in strength and elongation, as an evidence of SCC, in SSR tests performed with notched specimens in ethanol environments.

The FCG and SSR results show an important effect of the ethanol environment on the different regions of a welded API X70 steel pipe, demonstrating the efficiency of these experiments, which were implemented based on the developed methodology.

## Conclusions

The present methodology offers a detailed procedure for producing samples from circumferential welds on steel pipes and preparing specimens properly designed to promote environment-induced cracking in different regions of the welds.

Constructive details of an experimental apparatus, suitable for performing SSR and FCG tests in ethanol medium, are presented so that experiments can be easily replicated.

A previous paper [5], as well as additional results, validated the present methodology for SCC evaluation of circumferential welds, such as those used to construct of pipelines.
